# Mycotic aneurysm of the popliteal artery secondary to *Streptococus pneumoniae*: a case report and review of the literature

**DOI:** 10.1186/1752-1947-3-117

**Published:** 2009-11-10

**Authors:** Shane D Killeen, Noel O'Brien, Martin J O'Sullivan, George Karr, H Paul Redmond, Gregory J Fulton

**Affiliations:** 1 Departments of General Surgery, Cork University Hospital, Wilton, Cork, Ireland; 2 Neurosurgery, Cork University Hospital, Wilton, Cork, Ireland

## Abstract

**Introduction:**

Cases of true mycotic popliteal artery aneurysm are rare. Presentation is variable but invasive and non-invasive investigations collectively facilitate diagnosis and guide operative procedures. Definitive treatment generally utilizes surgical intervention with excision and reconstruction using autologous vein graft. Prolonged targeted antibiotic therapy is an important adjuvant.

**Case presentation:**

We describe the clinical presentation, radiological investigations and strategies on the management of a 47-year-old Caucasian Irish man who presented with a mycotic aneurysm of the popliteal artery due to thromboembolisation from *Streptococus pneumoniae *endocarditis.

**Conclusion:**

Cases of true mycotic popliteal artery aneurysms are rare. To the best of our knowledge this is the first documented case of a popliteal artery mycotic aneurysm developing secondary to *Streptococus pneumoniae *highlighting the changing profile of causative microorganisms.

## Introduction

True mycotic popliteal artery aneurysm is a rare condition and its presentation is variable. Definitive treatment is generally characterised by excision and reconstruction using an autologous vein graft. We describe the clinical presentation, radiological investigations and management strategies employed in the case of a 47-year-old man who presented with a mycotic aneurysm of the popliteal artery (MPAA) secondary to thromboembolisation from *Streptococus pneumoniae *endocarditis.

## Case presentation

A 47-year-old Caucasian Irish man was admitted to our intensive care unit (ICU) with multiple cerebral infarcts secondary to septic emboli from *Streptococus pneumoniae *endocarditis vegetations. After a prolonged ICU course, the patient was eventually transferred to the ward with minimal neurological dysfunction. The patient, however, developed pain in his right calf 6 weeks after admission.

**Figure 1:**
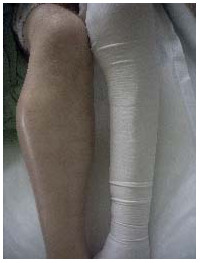
**Tender, pulsatile mass in the right upper calf consistent with a popliteal aneurysm**.

A clinical examination revealed a pyrexic patient with a swollen, pulsatile and tender right upper calf mass, palpable distal pedal pulses and foot drop (Figure 1). A mycotic aneurysm of his right popliteal artery (MPAA) was visible on computer tomography (CT) angiogram. Sepsis-related renal impairment precluded formal contrast angiography. Magnetic resonance angiography confirmed the presence of a large saccular aneurysm of the right below-knee popliteal artery approximately 8 × 9.5 × 8 cm, extending to the trifurcation with satisfactory inflow and outflow vessels.

**Figure 2:**
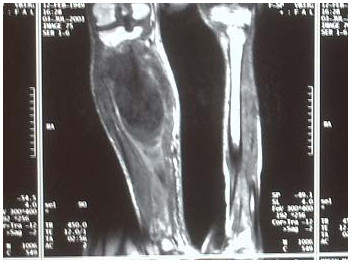
**Magnetic resonance image scan demonstrating a saccular aneurysm of the right below-knee popliteal artery**.

The contralateral popliteal artery was normal (Figure 2). Urgent operative repair involved aneurysm excision and reconstruction using reversed ipsilateral long saphenous vein.

**Figure 3:**
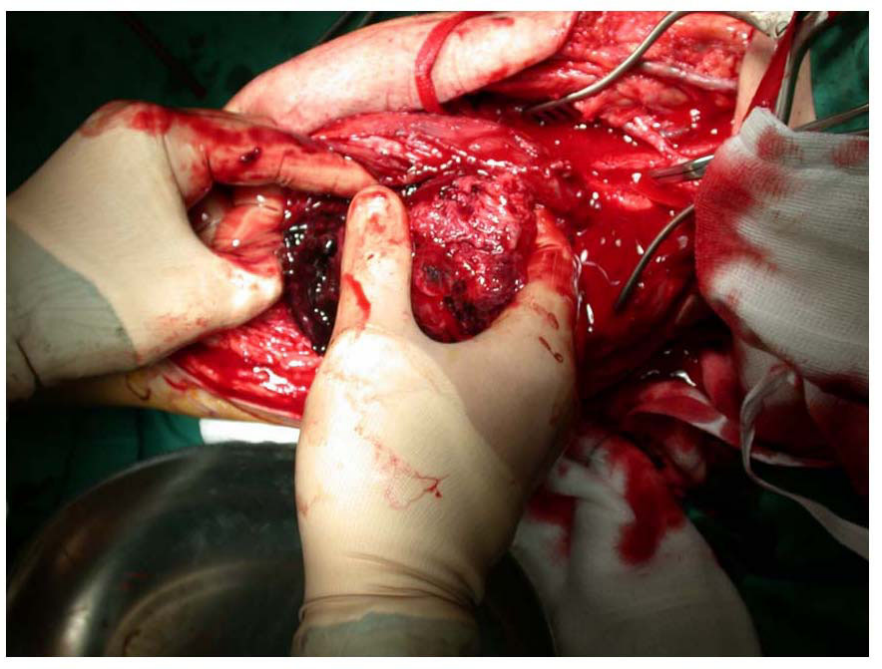
**Aneurysm of the below-knee popliteal artery with proximal control and evacuation of intraluminal thrombus**.

A curved incision over the right popliteal fossa revealed a saccular aneurysm of the below- knee popliteal artery involving the trifurcation. Proximal control was attained and the aneurysm opened, exposing a significant amount of haematoma and thrombus (Figure 3). Samples were sent for histological and microbiological assessment. The distal tibioperoneal trunk was identified and a reversed vein graft was interposed between it and the below-knee popliteal artery. After the operation, pulses were present in the posterior tibial and peroneal arteries. The patient's lower limb sensory deficit improved immediately and the foot drop resolved slowly.

**Figure 4:**
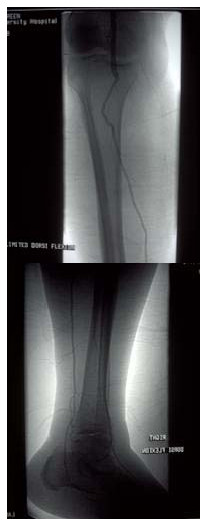
**Right femoral angiogram demonstrating a patent vein graft extending from the distal popliteal artery to the posterior tibial artery with retrograde filling of the anterior tibial artery via the planter arch**.

A femoral angiogram performed 2 weeks after the procedure demonstrated satisfactory graft segment flow (Figure 4). No organism was identified on microbiological assessment and atherosclerosis was not evident on histological examination. The patient started physiotherapy. Three months after presentation, he could already move on his own and had normal ankle brachial indices.

## Discussion

According to Wilson's widely held classification, mycotic aneurysms are strictly defined as "infected aneurysms developing in a previously normal artery secondary to septic embolisation due to bacterial endocarditis" [1]. Thus, although the Dublin surgeon Jolliffe Tufnell reported the first case of a ruptured infective popliteal aneurysm in 1885 [2], it was Stengel and Wolferth who reported the first "mycotic" popliteal artery aneurysm (MPAA) in 1923 [3].

To date there are fewer than 50 cases of "true" MPAA reported in the literature. The estimated male-to-female ratio is 11:3 with an age range of 2 to 81 years with the mean age of occurrence at 41 years old [1]. This contrasts with the results of other examinations on the causes of popliteal aneurysms (49:1, 50-80 years and 61 years, respectively) [4-8]. MPAA is a consequence of septic embolisation, usually from bacterial endocarditis, whereby emboli are lodged in the lumen or vaso vasorum of normal or abnormal peripheral arteries. This leads to vessel wall infection and ischemia resulting in medial destruction and aneurysm formation [3]. The normal intima is very resistant to infection and, therefore, healthy arteries are rarely affected unless the organism is very virulent or the patient is immunocompromised. In less than 50% of reported cases, the causative organism can be identified from operative specimens as a result of pre-operative antibiotic therapy [5]. Cases of exotic organisms are getting more isolated, which probably reflects an aging and immunocompromised population and increased use of prosthetic heart valves (Table 1).

**Table 1:** Organisms cultured from mycotic popliteal aneurysms collected through a review of the English language literature

*Staphylococcus aureus*	5 [5]
*Streptococcus viridans*	3 [7]

*Staphylococcus epidermis*	2 [5]

*Campylobacter jejuni*	2 [12,13]

*Streptococcus faecalis*	1 [6]

*Streptococcus pneumoniae*	1*

*E. coli*	1 [5]

*Tuberculosis*	1[14]

*Salmonella *spp	1 [15]

Culture negative	17

*This case.

MPAA customarily presents as a painful, tender, pulsatile leg swelling in pyrexic patients with a definitive or unsuspected infective focus [7]. Symptoms and signs of ischemia are often evident, secondary to thrombosis or rupture, which is a particular complication of popliteal aneurysms. Presentation may also mimic a deep vein thrombosis [6].

Although an estimated 3% of non-mycotic popliteal artery aneurysms can produce neurological symptoms and signs, possibly due to direct compression or occlusion of the vasonervorum arising from the popliteal artery [9], our patient's condition is one of the few documented cases of MPAA that produced a definitive neurological defect.

Laboratory studies are unhelpful in diagnosing MPAA and often indicate a non-specific leukocytosis or increased erythrocyte sedimentation rate (ESR) and C-reactive protein (CRP). Blood cultures are positive in only 50% of reported cases, but negative blood cultures and Gram stains do not necessarily rule out a mycotic aneurysm (Table 2).

**Table 2:** Presentation of mycotic popliteal aneurysms collected through a review of English language literature

Swelling in the popliteal fossa	33 (100%)
Fever	28 (85%)

Elevated erythrocyte sedimentation rate/C-reactive protein	25 (77%)

Leucocytosis	24 (72%)

Ischemia	12 (37%)

Deep vein thrombosis	5 (14%)

Rupture	2 (6%)

Foot drop	1 (3%)*

*This case.

Non-invasive procedures such as colour-duplex ultrasonography, CT and magnetic resonance imaging (MRI) or angiogram can establish a diagnosis of postoperative alopecia areata (PAA) and provide information regarding the size, diameter, morphology (saccular morphology being suggestive of an infective aetiology) and its relationship to surrounding structures [5].

Angiography, either conventional or digital subtraction, is important to demonstrate the status of the inflow and outflow vessels, hence guiding any operative approach [4,5].

CT and MRI as vessel imaging alternatives can be satisfactory, as in this case where the patient's temporary sepsis-related renal impairment precluded formal contrast angiography [5].

Ankle brachial index (ABI) measurements provide an objective, measurable and repeatable index of ischemia. Broad-spectrum antibiotics should be commenced pre-operatively and continued for a variable period postoperatively. If no causative pathogen is identified, at least two synergistic agents should be employed. Since there are no clear guidelines regarding the duration of postsurgical therapy, a 6-week oral course seems to be a prudent approach [5,10].

MPAA generally mandates resection and revascularisation [4-8,10]. The techniques and anatomic approaches in treating mycotic popliteal aneurysms can be different from those employed for their non-mycotic counterparts. There is no consensus in the literature with regards to approach, with medial and posterior approaches being equally utilized [4,5,10]. Infective aetiology prohibits the use of prosthetic conduits to restore flow and autologous long saphenous vein grafts were the overwhelming conduit of choice, either as *in situ *vessels or reversed superficial vein from the ipsilateral or contralateral lower (or upper) limb [5,10]. Deep leg veins, however, may be a viable alternative to long saphenous vein [11]).

The literature includes only a few reports of amputation, failure of reconstructive procedure and the need for secondary reconstruction or amputation, which probably reflects an unwillingness to publish perceived treatment failures. It is thus reasonable to assume a total limb salvage rate, a 5-year patency rate and a primary and/or secondary amputation rate that are at least comparable with, if not worse than, those of non-mycotic popliteal aneurysms (in the region of 60-72%, 50-86%, and 18-27.5% and/or 28-40%, respectively [5]).

## Conclusion

Mycotic popliteal artery aneurysm is a rare but potentially devastating condition. A high index of suspicion is therefore necessary. Invasive and non-invasive investigations facilitate diagnosis and guide operative procedures. Surgical intervention with excision and reconstruction using an autologous vein graft is the method of choice. Prolonged antibiotic therapy should initially be broad spectrum until pathogen identification can allow targeted therapy.

## Abbreviations

ABI: ankle brachial index; CT: computer tomography; ESR: erythrocyte sedimentation rate; CRP: C-reactive protein; ICU: intensive care unit; MPAA: mycotic aneurysm of the popliteal artery; PAA: postoperative alopecia areata;

## Consent

Written informed consent was obtained from the patient for publication of this case report and any accompanying images. A copy of the written consent is available for review by the Editor-in-Chief of this journal.

## Competing interests

The authors declare that they have no competing interests.

## Authors' contributions

SK retrieved all clinical data and conducted the literature review. NOB and MOS supplied the radiological images and also contributed to the literature review. HPR and GK were major contributors to the overall writing of the manuscript. GF contributed to the literature review, performed the procedure described, supplied the clinical images and conducted the specimen analysis. All authors read and approved the final manuscript.
